# Assessment of perceived dengue risk and prevention practices among youth in Bangladesh

**DOI:** 10.1038/s41598-024-54238-y

**Published:** 2024-02-16

**Authors:** Abu Bakkar Siddique, Nishat Tamanna Omi, Sayed Mohammad Rasel, Sayeda Samira Binte Hoque, Naimur Rahman, Sanjida Sarker, Ankon Ghosh, Imtiaz Ahmed, Yeasin Akash, Ayesha Ahmed, Md. Tajuddin Sikder

**Affiliations:** 1 Department of Public Health and Informatics, Jahangirnagar University, Savar, Dhaka, 1342 Bangladesh; 2 Centre for Advanced Research Excellence in Public Health, Savar, Dhaka, 1342 Bangladesh; 3 International Centre for Research, Innovation, Training and Development (ICRITD), Dhaka, Bangladesh; 4 AMR Reference Laboratory (Research), Bangladesh Livestock Research Institute, Savar, Dhaka, 1341 Bangladesh; 5 Health and Environmental Epidemiology Laboratory (HEEL), Jahangirnagar University, Savar, Dhaka, 1342 Bangladesh

**Keywords:** Dengue, Perceived risk, Prevention, Practices, Youth, Bangladesh, Viral infection, Medical research, Risk factors

## Abstract

Dengue fever is a global public health concern, especially in countries like Bangladesh. This study examines youth perceived dengue risk, and preventive practices, providing valuable insights into their behavior regarding this mosquito-borne disease. A cross-sectional survey was undertaken in various regions of the Dhaka district in Bangladesh. Face-to-face interviews were conducted with 1,358 participants using convenience sampling, spanning the period from September 2 to October 10, 2023. A semi-structured questionnaire covered informed consent, socio-demographic data, and questions about perceived dengue risk scale (12 items), and prevention practice (13 items). Participants’ mean age was 22.02 ± 1.58 years. The average scores for perceived dengue risk, and prevention practices were found to be 51.39 ± 12.01 (out of 96), and 55.57 ± 14.55 (out of 104) respectively. Previous dengue history, history of other vector-borne diseases, gender, educational level, father's educational qualification, employment status, adequate sleep duration, father's occupation, etc. are factors associated with higher risk and prevention practices regarding dengue. The research underscores the importance of tailoring prevention strategies for different demographics, raising awareness, and promoting active engagement in preventive measures. These insights are crucial for developing effective public health policies and campaigns to combat dengue.

## Introduction

Public health faces a significant threat from dengue, a well-documented global health issue^1^. The primary vectors of this mosquito-borne disease, which afflicts 129 countries, are *Aedes* mosquitoes, notably *Aedes aegypti* and *Aedes albopictus*, responsible for transmitting dengue through their bites^2,3^. Dengue, caused by four known DENV serotypes (DEN-1, 2, 3, 4)^4^, can lead to four distinct manifestations in humans. According to the World Health Organization (WHO), these include mild, with warning signs, and severe forms, resulting in a wide spectrum of illnesses, ranging from mild flu-like symptoms to subclinical disease^5,6^.

Globally, WHO reports between 50 and 100 million dengue cases each year, with approximately 20,000 documented deaths^2,7^. South Asia, including Bangladesh, serves as a hotspot for several infectious diseases, including dengue fever (DF)^8^. Asian countries, especially South-East Asia, where the first dengue viral infection was identified, account for nearly 70% of dengue cases^8^. Bangladesh's subtropical, humid climate provides an ideal environment for the dengue vector, promoting its increased transmission^9^. The country's first dengue outbreak was documented in 1964, with a significant surge in cases observed in 2019, particularly in Dhaka, responsible for over half of all national dengue cases^3^. In 2019, Bangladesh experienced its most severe dengue incidence to date, with 112,000 cases and 129 fatalities^10^. As of August 27, 2023, a total of 119,133 cases and 569 deaths had been reported from 64 districts in Bangladesh this year^11^. Furthermore, alterations in meteorological conditions, rainfall patterns, and humidity contribute to the prevalence of dengue, posing a significant public health concern^12^. This underscores the critical need for dengue prevention and control efforts in the country.

Dengue presents a threat to approximately half of the world's population, impacting individuals across almost all age groups including young individuals^8,13^. Ensuring personal safety and effectively managing mosquito populations are pivotal in disease prevention, with a primary focus on avoiding mosquito bites^6^. Regrettably, a significant portion of the population remains unaware of these preventive measures and neglects to follow essential guidelines or take proactive steps, contributing to the rising incidence of dengue infections^2^. Consequently, dengue prevention practices are of utmost importance, especially in the context of Bangladesh^14^. Furthermore, the youth (15 to 24 years of age) of Bangladesh constitute a critical demographic group^8,15^. With a substantial portion of the nation's population falling within the youth category, the future of the country lies in their hands. These young individuals will assume roles as future decision-makers, scientists, policymakers, and advocates, significantly influencing the nation's response to infectious diseases^16^. Additionally, a study of national surveillance data from 2011 to 2015 revealed that the most vulnerable group was the youth, primarily students^17^. Therefore, it is imperative to understand their perceived dengue risk and current prevention practices.

The aim of this research is to investigate the level of perceived dengue risk, prevention practices, and the factors associated with these among youth in Bangladesh. To date, there is no single study on perceived dengue risk and prevention practices among youth in Bangladesh. The study's findings will not only contribute to our understanding of dengue-related behaviors and attitudes among the youth but also offer practical recommendations for public health authorities, policymakers, and non-governmental organizations working to combat the disease in Bangladesh. Through a comprehensive analysis of the perceived dengue risk and prevention practices among the youth, who often serve as influential agents of behavioral change, this research can contribute to more effective and tailored strategies to combat dengue.

## Materials and methods

### Study area

Data were gathered through face-to-face interviews as part of a cross-sectional survey conducted in various areas of the Dhaka district, Bangladesh, between September and October 2023.

### Sample size

The sample size was calculated using the following equation:

$$n=\frac{{z}^{2}pq}{{d}^{2}}; n=\frac{{1.96}^{2}\times 0.5\times \left(1-0.5\right)}{{0.05}^{2}}=384.16\approx 384$$

here *n* = number of samples, *z* = 1.96 (95% confidence level), *p* = prevalence estimate (50% or 0.5), as there is no previous study in Bangladesh, *q* = (1-*p*), *d* = Precession of the prevalence estimate (10% of 0.5).

Anticipating a prevalence estimate (p) of 50% in our study, we initially calculated a sample size of 423 individuals, accounting for a 10% non-response rate. However, to enhance the study's robustness, we surpassed this estimate by recruiting a total of 1358 participants.

### Study design, participants, and procedure

The current study utilized a cross-sectional survey design based on face-to-face interview, conducted between September 2 and October 10, 2023. Our study participants were young people (15 to 24 years of age)^15^. Participants were recruited through a non-probability sampling method (convenience sampling), with each interview lasting around 10–15 min. A total of 1410 individuals initially participated in the surveys, but incomplete responses were excluded, resulting in a final dataset of 1358 surveys. Data collection utilized a paper-based semi-structured questionnaire in Bangla (the participant's native language) at their current residential locations (community level). Given the sensitivity of the topic, trained research assistants exclusively conducted data collection, ensuring strict confidentiality.

A preliminary assessment involving 30 participants from the designated population was conducted to evaluate the questionnaire's acceptability and transparency. Based on the pilot test results, some minor modifications were made to the questionnaire; however, these adjustments were not included in the final analysis. The initial page of the questionnaire featured an informed consent statement elucidating the study's objectives, procedures, and the participant's autonomy to decline participation. Prior to commencing the survey, participants were explicitly asked for their voluntary and spontaneous consent with the query, "*Are you willing to participate in this study voluntarily and willingly*?". The inclusion criteria of the participants included: i) young people (15 to 24 years of age)^15^, ii) ability to talk and read Bengali, iii) living in Bangladesh and Bangladeshi residents, and iv) willingness to participate in the study. The participants below 18 years and more than or equal to 25 years and unwillingness to participate were excluded at the time of the interview.

### Measures

#### Socio-demographic measures

Demographic details were collected through inquiries covering various aspects, including age, educational attainment (below university/university level), residence type (rural/urban/semi-urban), monthly family income (less than 20,000 BDT/20,000 to 30,000 BDT/more than 30,000 BDT) [BDT = Bangladeshi Taka, 1 BDT = 0.0091 USD]^18^, gender (male/female), marital status (married/unmarried), family structure (nuclear/large), employment status (employed/unemployed), prior history of dengue (yes/no), family history of dengue (yes/no), previous exposure to vector-borne diseases (yes/no), average sleep duration (less than 7 h/7 to 9 h/more than 9 h), daily social media usage in hours (less than 2 h/2 to 4 h/more than 4 h), father's occupation (job holder/business/others/unemployed), father's educational qualification (primary or below/secondary or higher secondary/university level), and self-perception of mental health (good/bad).

#### Perceived dengue risk

The Perceived Dengue risk scale is a tool used to measure an individual's perception of their risk to Dengue, consisting of 12 items. For example, questions used in the scale like: ‘‘*I am at risk to get dengue fever, Dengue fever is a seasonal disease, I will be safe from it if the dengue season has passed, I am bitten by mosquitoes every day, but I have never been infected with dengue fever. So, I am not at risk of getting dengue fever, *etc.’’ with eight possible answers. It is used in research and clinical settings to identify individuals with perceived Dengue risk/ threat and inform targeted interventions^19^. The scale is scored on a Likert scale with the possible response of between 1 and 8 (i.e., *1 strongly disagree–8 strongly agree* "]) and helps tailor prevention strategies and increase awareness. The possible total scores range from 12 to 96. Higher scores indicate a stronger perception dengue risk. The overall score is derived by summing the scores of each item. It should be used with other assessments for a comprehensive understanding of risk. In the current investigation, this scale was shown to have extremely good reliability (Cronbach's alpha = 0.91).

#### Dengue prevention practices

To document the prevention practices status, the participants were asked thirteen questions (e.g., *“I use mosquito repellent (lotion/spray/coil), I always keep water containers in my house tightly closed, I check for potential mosquito breeding inside the house, I put larvicide into the water storage to kill the mosquito larvae.”* (see details in Table 1) with eight-point Likert scale (i.e., *1 strongly disagree–8 strongly agree* "). These questions were taken from a previous validated study^19^. The total score was obtained by summating the scores of all items and ranges from 13–104, with a higher score indicating a higher level of prevention practices. The Cronbach Alpha of attitudes items were 0.84. The skewness and kurtosis of the total scores were between ± 2.

**Table 1:** Dengue-prevention practice-related question.

Items	Mean ± Sd
1. I use mosquito repellent (lotion/spray/coil)	4.50 ± 2.12
2. I always keep water containers in my house tightly closed	4.78 ± 1.80
3. I check for potential mosquito breeding inside the house	3.97 ± 1.72
4. I put larvicide into the water storage to kill the mosquito larvae	3.60 ± 1.68
5. I only dispose rubbish at the designated place	5.04 ± 1.69
6. I made complaint to the authority when I found an illegal dumping site	4.05 ± 1.72
7. I keep my drainage system properly maintained	4.63 ± 1.72
8. I do not keep unused items that can store water	4.85 ± 1.71
9. I made complaint to the authority when there is damaged vehicle idling in my neighborhood	4.00 ± 1.67
10. I check for potential mosquito breeding place around the neighborhood	4.21 ± 1.68
11. I participate in gotong royong activities to prevent dengue	4.03 ± 1.70
12. I made complaint to the authority when I found illegal garden	3.96 ± 1.68
13. I made complaint to the authority when I found illegal building structure	3.95 ± 1.75

### Statistical analysis

The data underwent analysis utilizing Statistical Package for Microsoft Excel (version 2021), SPSS version 26.0 (Chicago, IL, USA), and STATA (version 15.0). Initial data processing, which involved cleaning, coding, and sorting, was executed with Microsoft Excel. Subsequently, the prepared Excel file was imported into SPSS for computation of descriptive statistics such as frequencies, percentages, means, and standard deviations. The final phase involved bivariate and multivariable linear regression analyses in STATA, considering the total scores of perceived dengue risk and prevention practices as the dependent variables. Significance for all analyses was set at a p-value less than 0.05.

### Ethics approval and consent to participate

The Bio-Safety, Bio-Security, and Ethical Committee at Jahangirnagar University thoroughly examined and approved the study protocol [Ref. No: BBEC, JU/M2023/08(59)]. All of the study's procedures conformed to standards for human involvement research (e.g., the Helsinki Declaration). Data were collected anonymously, and numerical codes were employed for analysis. Inform written consent was obtained from each participant where the study's procedures, objectives, and confidentiality about their information, etc. were clearly documented. The data were collected anonymously and analyzed using numerical codes and no identifying numbers or images were taken.

## Result

### General characteristics of the participants

The table presents a comprehensive overview of the study participants' demographics and lifestyle. The mean age is 22.11 years (SD 1.72), with an almost equal split in educational qualification between below university (50.2%) and university level (49.8%). Urban residents account for 47.2%, rural for 34.4%, and semi-urban for 18.4%. Monthly family income is predominantly between 20,000 to 50,000 BDT (51.5%). Gender distribution is balanced at 50.2% male and 49.8% female. The majority are unmarried (92.6%) and belong to nuclear families (50.5%). Employment status shows 82.7% unemployed and 17.3% employed. Health history indicates 24.2% with a previous Dengue history and 26.1% with a family history. 43.8% sleep less than 7 h, 51.8% use social media for more than 4 h, and 78.8% perceive their mental health as good. Father's occupation is varied, with 46.5% job holders, 33.3% involved in business, 16.6% in other occupations, and 3.6% unemployed. Father's educational qualifications include 12.1% with primary or below, 37.3% with secondary/higher secondary, and 50.6% with university-level education. This concise overview provides key insights for analyzing the relationships between demographics, lifestyle, and health factors in the studied population (Table 2).

**Table 2:** General characteristics of the population (N = 1358).

Variables	n (%)
Age (Mean ± SD)	22.11 ± 1.72
Educational qualification	
Below university	682 (50.2)
University level	676 (49.8)
Permanent residence	
Rural	467 (34.4)
Urban	641 (47.2)
Semi-urban	250 (18.4)
Monthly family income	
Less than 20,000 BDT	404 (29.8)
20,000 to 50,000 BDT	700 (51.5)
More than 50,000 BDT	254 (18.7)
Gender	
Male	682 (50.2)
Female	676 (49.8)
Marital status	
Unmarried	1257 (92.6)
Married	101 (7.4)
Family type	
Nuclear family	686 (50.5)
Large family	672 (49.5)
Employment status	
Employed	235 (17.3)
Unemployed	1123 (82.7)
Previous history of Dengue	
Yes	328 (24.2)
No	1030 (75.8)
Family history of dengue	
Yes	354 (26.1)
No	1004 (73.9)
Previous history of vector-borne disease except Dengue (Malaria, Filaria, West Lime virus, Lime disease, etc.)	
Yes	320 (26.1)
No	1038 (76.4)
Average sleeping time	
Less than 7 h	595 (43.8)
7 to 9 h (normal)	669 (49.3)
More than 9 h	94 (6.9)
Daily social media use (hours)	
Less than 2 h	38 (2.8)
2 to 4 h	616 (45.4)
More than 4 h	704 (51.8)
Father’s occupation	
Job holder	632 (46.5)
Business	452 (33.3)
Others	225 (16.6)
Unemployed	49 (3.6)
Father’s educational qualification	
Primary or below	164 (12.1)
Secondary/ higher secondary	506 (37.3)
University level	688 (50.6)
Self-perception about own mental health	
Good	1070 (78.8)
Bad	288 (21.2)

BDT Bangladeshi Taka, 1 BDT = 0.0091 USD in 4 November, 2023, *SD* Standard Deviation.

### Perceived dengue risk

The mean score of perceived dengue risk was 51.39 ± 12.01 out of 96, indicating an overall correct percentage of 53.53. As per the multiple linear regression analysis, the positively predicting factors of perceived dengue risk included: i) participants with an education level below university ((ꞵ = 0.14, *p* < 0.001) in reference to ‘university’, ii) being female (ꞵ = 0.08, *p* < 0.003) in reference to ‘male’, iii) previous history of dengue (ꞵ = 0.03, *p* < 0.029) in reference to ‘no’ previous history of dengue, iv) previous history of vector-borne disease (ꞵ = 0.04, *p* < 0.048) in reference to ‘no’ previous history of vector-borne disease, v) father's occupation-job holder (ꞵ = 0.15, *p* < 0.042 in reference to ‘unemployed’, vi) father’s educational qualification-illiterate/ primary level (ꞵ = 0.07, *p* < 0.020) in reference to ‘university level’ (Table 3).

**Table 3:** Regression analysis predicting perceived dengue risk.

Variables	Overall	Bivariate regression analysis					Multivariable regression analysis				
	Mean (SD)	B	SE	t	ꞵ	*p*-value	B	SE	t	ꞵ	*p*-value
Age		0.39	0.20	1.91	0.05	0.057	0.31	0.21	1.49	0.04	0.136
Education level											
Below university	53.07 (11.54)	3.36	0.64	5.20	0.13	** < 0.001**	3.49	0.68	5.11	0.14	** < 0.001**
University	49.71 (12.25)	Ref					Ref				
Permanent Residence											
Rural	50.82 (12.61)	Ref					Ref				
Urban	52.04 (11.31)	1.22	0.73	1.68	0.05	0.094	1.01	0.79	1.27	0.04	0.203
Semi-urban	50.83 (12.59)	0.01	0.94	0.01	< 0.01	0.990	0.09	0.95	0.10	< 0.01	0.918
Monthly family income											
> 20,000 BDT	50.66 (13.09)						Ref				
20,000–30,000 BDT	51.71 (11.86)	1.05	0.75	1.40	0.05	0.161	− .08	0.81	− 0.11	< 0.01	0.914
> 30,000 BDT	51.70 (10.85)	1.03	0.96	1.08	0.03	0.282	0.02	1.08	0.02	< 0.01	0.982
Gender											
Male	52.46 (12.10)						Ref				
Female	50.33 (11.85)	2.12	0.64	3.27	0.08	**0.001**	1.97	0.66	2.96	0.08	**0.003**
Marital status											
Unmarried	51.40 (11.86)	Ref									
Married	51.41 (13.85)	0.00	1.24	0.01	0.08	0.994	0.84	1.27	0.66	0.01	0.509
Family type											
Nuclear	51.31 (12.77)	Ref									
Large	51.49 (11.20)	0.18	0.65	0.28	0.08	0.782	0.80	0.67	1.20	0.03	0.231
Employment status											
Employed	50.63 (13.39)	Ref									
Unemployed	51.56 (11.71)	0.92	0.86	1.08	0.02	0.282	1.39	0.89	1.56	0.04	0.119
Previous history of Dengue											
Yes	52.76(12.43)	1.16	0.74	1.57	0.04	**0.018**	1.01	0.65	0.64	0.03	**0.029**
No	50.96(11.85)	Ref					Ref				
Family history of dengue											
Yes	52.26(11.57)	1.16	0.74	1.57	0.01	0.117	− **0.02**	0.80	− 0.03	< 0.01	0.980
No	51.09(12.16)	Ref					Ref				
Previous history of vector-borne disease											
Yes	52.73(11.01)	1.73	0.74	2.26	0.07	**0.024**	0.96	0.82	1.69	0.04	**0.048**
No	50.99(12.29)	Ref									
Average sleeping time											
Less than 7 h	51.29(11.41)	0.45	1.33	0.34	0.01	0.731	1.23	1.33	0.92	0.05	0.356
7 to 9 h	51.57(12.19)	0.74	1.32	0.56	0.03	0.575	1.11	1.32	0.84	0.04	0.399
More than 9 h	50.83(14.39)	Ref					Ref				
Daily social media usage											
Less than 2 h	52.08 (10.77)	0.71	2.00	0.36	< 0.01	0.720	0.57	1.98	0.29	< 0.01	0.773
2 to 4 h	51.39 (12.37)	0.03	0.66	0.05	< 0.01	0.961	− 0.02	0.66	0.04	< 0.01	0.965
More than 4 h	51.36 (11.78)	Ref					Ref				
Father’s occupation											
Job holder	51.75 (12.46)	4.77	1.77	2.68	0.19	**0.007**	3.69	1.81	2.03	0.15	**0.042**
Businessman	51.58 (11.33)	4.60	1.80	2.55	0.18	**0.011**	3.75	1.79	2.09	0.14	**0.037**
Others	50.99 (11.90)	4.00	1.89	2.12	0.12	**0.034**	4.32	1.89	2.29	0.13	**0.022**
Unemployed	46.98 (12.18)	Ref					Ref				
Father’s Educational qualification											
Illiterate/ primary level	48.52 (14.81)	3.53	1.07	3.29	0.14	**0.001**	2.74	1.17	2.33	0.07	**0.020**
Secondary/Higher Secondary	52.06 (11.57)	3.08	1.04	2.96	0.12	**0.003**	0.37	0.77	0.49	0.01	0.626
University level	51.60 (11.57)	Ref					Ref				
Self-perception about own mental health											
Good	51.35 (12.37)	Ref					Ref				
Bad	51.56 (10.61)	0.20	0.79	0.26	< 0.01	0.797	− 0.67	0.96	− 0.70	− 0.01	0.485

B = unstandardized regression coefficient; SE = Standard error; β = standardized regression coefficient; Bold indicates significant; ^†^F_(16,1341)_ = 3.36; p < 0.001, R^2^_Adj_ = 0.027.

### Dengue prevention practice

**Figure 1:**
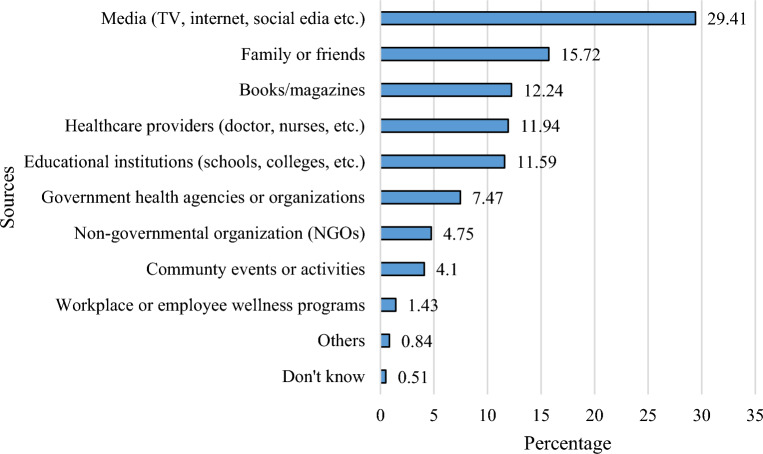
Sources of dengue prevention practice-related information.

The mean score of dengue prevention practice was 55.57 ± 14.55 out of 104, indicating an overall correct percentage of only 61.06%. As per as multiple linear regression analysis, the positively predicting factors of dengue prevention practice included: i) participants who are unemployed (ꞵ = 0.06, *p* < 0.025) in reference to ‘employed’, ii) previous history of dengue (ꞵ = 0.07, *p* < 0.042) in reference to ‘no’ previous history of dengue, iii) previous history of vector-borne disease (ꞵ = 0.09, p < 0.003) in reference to ‘no’ previous history of vector-borne disease, iv) sleeping time between 7 to 9 h (ꞵ = 0.12, *p* < 0.028) in reference to ‘more than 9 h’. v) father's educational qualification at university level (ꞵ = 0.03, *p* < 0.007) in reference to ‘Illiterate/ primary level’ (Table 4). Figure 1 illustrates the origins of information related to dengue prevention practices. Among the respondents, 29.41% and 12.24% cited media (including TV, internet, social media, etc.) and books/magazines, respectively, as their sources for information on dengue prevention practices.

**Table 4:** Regression analysis predicting dengue prevention practice.

Variables	Overall	Bivariate regression analysis					Multivariable regression analysis				
	Mean (SD)	B	SE	t	ꞵ	*p*-value	B	SE	t	ꞵ	*p*-value
Age		0.26	0.24	1.05	0.02	0.292	0.22	0.26	0.86	0.02	0.392
Education level											
Below university	56.10 (14.70)	1.06	0.78	1.34	0.03	0.179	1.52	0.83	1.83	0.05	0.068
University	55.04 (15.01)	Ref					Ref				
Permanent residence											
Rural	54.79 (15.25)	Ref					Ref				
Urban	56.23 (13.80)	1.22	0.73	1.62	0.04	0.105	1.32	0.97	1.37	0.04	0.172
Semi-urban	55.34 (15.08)	.55	1.14	0.48	0.01	0.629	0.45	1.16	0.39	0.01	0.696
Monthly family income											
> 20,000 BDT	54.70 (15.55)						Ref				
20,000–30,000 BDT	56.12 (14.46)	1.41	0.90	1.56	0.04	0.119	0.24	1.00	0.25	< 0.01	0.804
> 30,000 BDT	55.43 (13.08)	0.73	1.16	0.63	0.01	0.531	− 0.83	1.32	− 0.63	− 0.02	0.528
Gender											
Male	55.54 (14.95)						Ref				
Female	55.61 (14.15)	0.06	0.79	0.09	< 0.01	0.930	− 0.14	0.81	− 0.18	< 0.01	0.858
Marital status											
Unmarried	55.47 (14.41)	Ref									
Married	56.77 (16.27)	1.29	1.50	0.86	0.02	0.389	2.21	1.55	1.42	0.03	0.155
Family type											
Nuclear	55.61 (14.98)	0.07	0.79	0.09	< 0.01	0.929	− 0.13	.082	− 0.17	< 0.01	0.867
Large	55.54 (14.11)	Ref									
Employment status											
Employed	53.80 (15.59)	Ref									
Unemployed	55.94 (14.31)	2.13	1.04	2.05	0.05	**0.041**	2.44	1.09	2.24	0.06	**0.025**
Previous history of Dengue											
Yes	55.29 (14.42)	0.36	0.89	0.40	0.01	0.691	2.38	1.17	2.04	0.07	**0.042**
No	55.66 (14.60)	Ref					Ref				
Family history of dengue											
Yes	55.63 (14.06)	0.07	0.74	0.09	< 0.01	0.930	− 0.11	0.98	0.26	< 0.01	0.909
No	55.55 (14.73)	Ref					Ref				
Previous history of vector-borne disease											
Yes	57.21 (12.97)	2.13	0.92	2.30	0.06	**0.021**	3.42	1.13	3.01	0.09	**0.003**
No	55.07 (14.98)	Ref					Ref				
Average sleeping time											
Less than 7 h	55.31 (14.67)	2.72	1.61	1.69	0.09	0.091	2.79	1.63	1.71	0.09	0.087
7 to 9 h	56.22 (14.19)	3.63	1.60	2.27	0.12	**0.023**	3.56	1.61	2.21	0.12	**0.028**
More than 9 h	52.59 (16.05)	Ref					Ref				
Daily social media usage											
Less than 2 h	56.76 (15.05)	1.10	2.42	0.46	0.01	0.648	1.01	2.42	0.42	0.01	0.676
2 to 4 h	55.40 (15.25)	− 0.25	0.80	− 0.32	< 0.01	0.751	− 0.15	0.81	− 0.19	< 0.01	0.849
More than 4 h	55.66 (13.91)	Ref					Ref				
Father’s occupation											
Job holder	55.58 (14.50)	1.39	2.15	0.65	0.19	0.518	− 0.49	2.21	− 0.22	− 0.01	0.822
Businessman	55.06 (13.85)	0.87	2.18	0.40	0.18	0.689	− 0.39	2.19	− 0.18	− 0.01	0.859
Others	56.88 (15.81)	2.69	2.29	1.17	0.12	0.241	2.35	2.31	1.02	0.06	0.308
Unemployed	54.18 (15.52)	Ref									
Father’s Educational qualification											
Illiterate/ primary level	52.76 (16.61)	Ref					Ref				
Secondary/Higher Secondary	55.42 (14.46)	2.65	1.30	2.04	0.08	**0.042**	2.86	1.37	2.09	0.08	**0.037**
University level	56.36 (14.02)	3.60	0.26	2.85	0.12	**0.004**	3.89	1.44	2.70	0.03	**0.007**
Self-perception about own mental health											
Good	55.67 (14.85)	0.47	0.96	0.49	0.01	0.624	0.31	1.18	0.27	< 0.01	0.789
Bad	55.20 (13.42)	Ref					Ref				

B = unstandardized regression coefficient; SE = Standard error; β = standardized regression coefficient; Bold indicates significant; ^†^F_(18,1341)_ = 1.98; p < 0.001, R^2^_Adj_ = 0.011.

### Predicting the association between perceived dengue risk and dengue prevention practice

As per as multiple linear regression analysis, better dengue prevention practice is positively associated with higher perceived dengue risk: (ꞵ = 0.42, *p* < 0.001) (Table 5).

**Table 5:** Regression analysis predicting perceived dengue risk and dengue prevention practice.

Variables	Dengue risk				
	B	SE	t	ꞵ	*p*-value
Dengue prevention practice	0.34	0.02	17.18	0.42	< 0.001

B = unstandardized regression coefficient; SE = Standard error; β = standardized regression coefficient; Bold indicates significant; ^†^F_(1,1356)_ = 295.04; p < 0.001, R^2^_Adj_ = 0.178.

## Discussion

Assessing youth perceptions of dengue risk in Bangladesh is crucial for public health. Dengue, a globally concerning mosquito-borne virus, is on the rise in Bangladesh^20^. Given the substantial youth population, their outlook on dengue risk and preventive measures is of great importance. Their insights facilitate early detection and targeted prevention efforts^2^. This study delves into youth practices in response to perceived dengue risk, potentially guiding effective prevention measures^21^. Involving youth in dengue prevention can have a positive impact on communities^22^. The study's findings inform public health policies, addressing an existing research gap and making a significant contribution to the fight against dengue, shaping evidence-based strategies^23^.

This research revealed a significant correlation between the perception of dengue risk and several factors, including educational level, gender, previous dengue experience, history of other vector-borne diseases, fathers' occupational status, and fathers' educational qualifications. Notably, individuals with educational backgrounds below the university level exhibited a higher perceived risk of dengue compared to those with university-level education, a finding consistent with a study in Riohacha, Colombia^24^. This connection can be attributed to the fact that individuals with lower educational levels, particularly among the youth, often possess limited health literacy, making it challenging for them to grasp the risks associated with dengue and how to safeguard themselves^25^. This discovery will contribute to enhancing dengue risk awareness through increased education and awareness, aligning with the goals of our study (Table 1).

The study's findings indicate that female participants perceive a higher level of dengue risk compared to male, which is consistent with the results of another study^26^. This difference in perception may be attributed to females generally being more susceptible to fear and risk compared to male^27^. A study conducted in Bangladesh using national surveillance data similarly identified that the risk and mortality associated with dengue are elevated among females^28^. Additionally, in certain Asian communities, women tend to delay seeking hospital care until the later stages of the diseas^26^. However, it's worth noting that contrasting results were observed in a separate study^29^, possibly due to differences in the age groups of the participants. People with a prior history of dengue found to perceive a higher risk of contracting dengue, a finding in alignment with two studies that have suggested an association between previous exposure to different serotypes of the dengue virus and the perception of dengue risk^24,30^. This connection is likely influenced by personal experiences, as those who have had direct encounters with dengue or have close connections with individuals affected by the disease are more inclined to believe that the risk of dengue is elevated in their area^24,25^.

The study finding illuminated that there is a positive correlation between a prior history of vector-borne diseases and the perception of heightened dengue risk. This observation is consistent with findings from other studies^25,31^. The rationale behind this connection is that individuals who have encountered other vector-borne diseases may recognize the shared transmission method, often via mosquito bites, which subsequently leads them to believe there is a greater risk of contracting dengue^25^. Moreover, the current fluctuations in meteorological patterns, rainfall, and humidity can influence the risk of vector-borne diseases^32^. Interestingly positive association was found between father's occupation level with perceived dengue risk. Individuals whose fathers were employed were found to have a significantly higher risk compared to those whose fathers were unemployed. This findings aligns with another study^2^.The reasons behind the result of this association could be that occupational status can impact access to healthcare services, including early diagnosis and treatment of dengue cases which ultimate make them aware about dengue^33^.

This is evident from this study that, individuals whose fathers have only completed primary education or are entirely illiterate show a stronger correlation with perceived dengue risk and prevention practice compared to those with educated fathers. A similar outcome was observed in a Nepalian study^34^. Illiterate parents may possess limited knowledge regarding the significance of measures like using mosquito nets, repellents, or maintaining a clean environment to prevent dengue^35,36^. Additionally, the lack of education can act as a hindrance to accessing healthcare services, potentially resulting in delayed diagnosis and treatment of dengue cases^2^.

Key factors affecting dengue prevention practices encompass employment status, previous dengue or vector-borne disease history, sleep duration, and fathers' educational qualifications. Notably, individuals who are unemployed tend to exhibit more robust dengue prevention practices, a pattern corroborated by previous studies^36,37^. This might be attributed to the fact that individuals without jobs often spend more time at home, where they are more inclined to engage in cleaning and maintaining their living environments^38,39^.

Study findings demonstrates that who had previously contracted dengue fever tended to engage in more extensive dengue prevention practices. This discovery aligns with findings from other studies^40,41^. People who have experienced dengue in the past are typically more conscious of the disease's severity and the discomfort it brings. Those who have previously endured dengue may have an increased fear of experiencing the disease again, which in turn drives them to adopt preventive practices to lower their risk of reinfection^37,40^. There was a significant link between prior experience with vector-borne diseases and dengue prevention practices in this study, as seen in another studies^25,41^. Having had a previous illness can make individuals more inclined to use mosquito nets, repellents, and eliminate breeding sites, underlining the importance of prevention^31^.

A significant correlation was observed between the average duration of sleep and engagement in dengue prevention practices. Those who consistently get a recommended 7 to 9 h of sound sleep appear to be more conscious of dengue prevention measures, and this pattern was similarly identified in another study^42^. This connection may be attributed to the fact that individuals who enjoy better sleep tend to have improved overall health, which in turn enhances their ability to participate in activities such as eliminating stagnant water or using mosquito nets to guard against dengue^43^. Additionally, the study highlights a positive association between the perception of dengue risk and the adoption of dengue prevention practices. This outcome aligns with a separate study^24^. It's natural for people who perceive a dengue risk to take preventive measures. This correlation can be explained by the fact that heightened awareness of the disease and its potential consequences motivates individuals to actively engage in dengue prevention^31^.

In summary, this study offers valuable insights into the extent and factors linked to the perceived risk of dengue and preventive practices among the youth in Bangladesh, representing the primary objectives of our research. The findings align with previous research and offer new insights into the relationship between perceived risk and preventive measures. These results can guide focused public health interventions and campaigns to enhance awareness, motivating individuals to proactively engage in dengue prevention—our primary objective. Further research in this domain is crucial to bolster the evidence base and refine strategies for effectively combating the dengue virus.

### Strengths and limitations of this study

This study's primary strength is its comprehensive exploration of perceived dengue risk and preventive practices, utilizing a substantial sample size. This research represents a pioneering effort of its kind among the youth in Bangladesh. The findings provide valuable insights for policymakers, aiding in the development of effective plans to address both dengue outbreaks and preventive measures. The study acknowledges several limitations that warrant attention. Firstly, the reliance on convenience sampling may introduce selection bias, limiting the generalizability of findings to broader populations in different areas. Secondly, the collected data is susceptible to recall bias, response bias, and social desirability bias, potentially compromising the accuracy of responses due to reliance on self-reported measures. Additionally, the cross-sectional design hinders the establishment of causal relationships and a nuanced understanding of changes over time. A longitudinal or prospective study would be beneficial in this regard. Furthermore, the absence of a comparison group and limited generalizability to other regions or countries constrains the applicability of the findings. It is crucial to bear these limitations in mind when interpreting results and making conclusions.

## Conclusion

In conclusion, this study has provided a comprehensive analysis of perceived dengue risk and prevention practices among the youth in Bangladesh. The findings underscore high perceived risk and a significant gap in to preventive practices, revealing key influencers such as previous dengue history, history of other vector-borne diseases, gender, educational level, father's educational qualification, employment status, adequate sleep duration, and father's occupation. The study emphasizes the urgent need for targeted public health interventions to enhance awareness and motivate proactive engagement in dengue prevention, especially among the youth who play a pivotal role in shaping the nation's response to infectious diseases. The home message emphasizes the importance of developing effective plans by policymakers and non-governmental organizations to curb the escalating incidence of dengue in Bangladesh. This research contributes valuable insights to inform evidence-based strategies for combating the dengue virus effectively and safeguarding public health in the country, with a specific focus on the youth population in the country.

## Data Availability

Upon reasonable request, the corresponding author will share the data supporting this article.

## Abbreviations

DENV: Dengue virus
DF: Dengue fever
BDT: Bangladeshi Taka (currency)
WHO: World Health Organization
SPSS: Statistical Package for the Social Sciences
STATA: Software for Statistics and Data Science
Likert scale: A psychometric scale commonly involved in research surveys
Cronbach’s alpha: A measure of internal consistency reliability
IRB: Institutional Review Board
SD: Standard deviation
CI: Confidence interval
